# Bidirectional risk relationship between autoimmune blistering diseases and monoclonal gammopathies: A large-scale matched cohort study

**DOI:** 10.1016/j.jdin.2025.05.015

**Published:** 2025-06-21

**Authors:** Philip Curman, Binh T. Ngo, Ralf J. Ludwig, Michael Kasperkiewicz

**Affiliations:** a Dermato-Venereology Clinic, Karolinska University Hospital, Stockholm, Sweden; b Department of Medical Epidemiology and Biostatistics, Karolinska Institute, Stockholm, Sweden; c Dermatology and Venereology Division, Department of Medicine (Solna), Karolinska Institute, Stockholm, Sweden; d Lübeck Institute of Experimental Dermatology, University of Lübeck, Lübeck, Germany; e Department of Dermatology, Keck School of Medicine, University of Southern California, Los Angeles, California; f Institute and Comprehensive Centre for Inflammation Medicine, University-Hospital Schleswig-Holstein (UKSH), Campus Lübeck, Lübeck, Germany; g Department of Dermatology, UKSH, Campus Lübeck, Lübeck, Germany; h Division of Dermatology, Department of Medicine, David Geffen School of Medicine at University of California Los Angeles, Los Angeles, California

**Keywords:** autoimmune blistering diseases, cohort study, monoclonal gammopathy, pemphigoid, pemphigus, TriNetX

*To the Editor:* Both autoimmune diseases, including the potentially life-threatening immunobullous disorders pemphigus and pemphigoid, and monoclonal gammopathies (MG) share the common feature of uncontrolled production of pathologic immunoglobulins by over-reactive plasma cells (ie, autoantibodies and M proteins, respectively).^1^, ^2^, ^3^ The spectrum of MG mainly consists of a premalignant precursor condition known as MG of undetermined significance (MGUS) and a group of malignant hematologic diseases including multiple myeloma and Waldenström macroglobulinaemia.^4^ The association between autoimmune blistering disorders (AIBD) and MG has been only preliminarily studied.

To fill this knowledge gap, we conducted a large-scale, retrospective, propensity score-matched cohort study using the US Collaborative Network of the TriNetX platform,^5^ which provided access to 116,855,912 de-identified electronic health records from 68 US health care organizations at the time of data retrieval in February 2025. Cohorts and outcomes were defined by ICD-10CM codes. The main study cohorts were patients with AIBD (pemphigus or pemphigoid; *n* = 32,951) and MG (MGUS, multiple myeloma, or Waldenström macroglobulinemia; *n* = 303,152) matched 1:1 with controls. Controls were defined by a general health examination (Z00), and the diagnosis defined the index event. All individuals with the outcome prior to index were excluded during cohort retrieval. Outcomes for AIBD patients were MG (MGUS [D47.2], multiple myeloma [C90.0], and any MG [D47.2, C90.0 or C88.0]). For MG patients, the outcomes were AIBD (pemphigus [L10.0, L10.1, L10.2, or L10.4], BP [L12.0], any pemphigoid [L12], and any AIBD [L10 or L12]). Waldenström macroglobulinemia (C88.0) was included in the “any MG” category but was not analyzed separately due to insufficient power. Exclusion criteria included paraneoplastic pemphigus due to its well-established association with malignancies. The definitions and baseline characteristics of the study cohorts are detailed in Supplementary Tables I-III, available via Mendeley at https://data.mendeley.com/datasets/93px2tjyv4/1.

Compared to control subjects, pemphigus and pemphigoid patients had a significantly increased risk of developing any MG (hazard ratio [HR] 1.50, 95% confidence interval [CI] 1.18-1.92, *P* = .001), particularly MGUS (HR 1.64, 95% CI 1.25-2.15, *P* < .001) and within the first 1-3 years following the AIBD diagnosis (Fig 1, Supplementary Table IV, available via Mendeley at https://data.mendeley.com/datasets/93px2tjyv4/1), whereas MG generally did not meaningfully increase the likelihood of developing AIBD (Supplementary Table V, available via Mendeley at https://data.mendeley.com/datasets/93px2tjyv4/1). This risk was stable in sensitivity analyses including minimum baseline time window and documented survival criteria (Fig 2, Supplementary Table IV, available via Mendeley at https://data.mendeley.com/datasets/93px2tjyv4/1).

**Fig 1:**
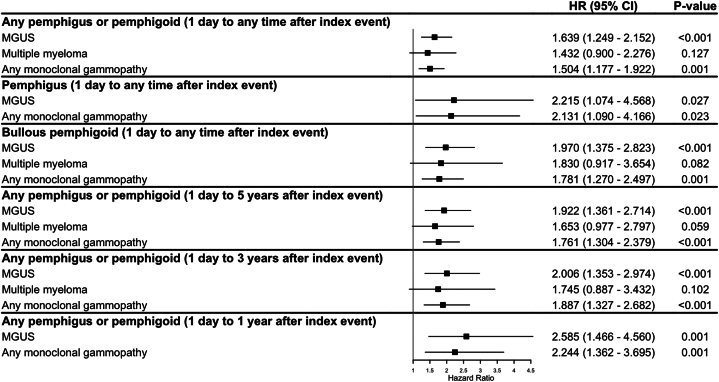
The risk of monoclonal gammopathy following a diagnosis of autoimmune blistering disease: Primary analyses. Outcomes with an unrestricted follow-up as well as 1, 3, and 5 years after index are displayed. Subgrouping highlighting the risk after a diagnosis of pemphigus and bullous pemphigoid are included. Overall risk elevations of any monoclonal gammopathy and monoclonal gammopathy of undetermined significance are shown. No results for multiple myeloma were displayed for some analyses due to the number of outcomes being too low to yield statistically reliable or interpretable estimates. *MGUS*, Monoclonal gammopathy of undetermined significance.

**Fig 2:**
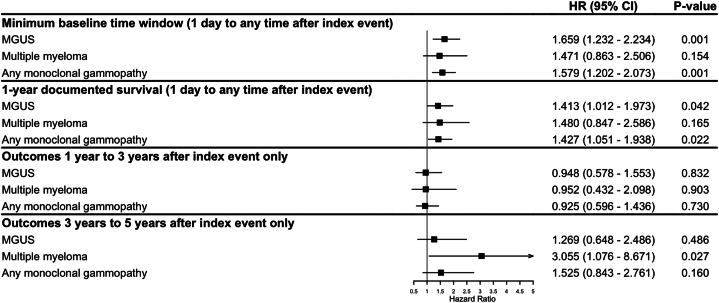
The risk of monoclonal gammopathy following a diagnosis of autoimmune blistering disease: Secondary analyses. Implementation of a minimum baseline time window and 1-year documented survival for unrestricted follow-up as well as outcomes exclusive to 1-3 and 3-5 years after index are shown. *MGUS*, Monoclonal gammopathy of undetermined significance.

The data from this so far largest epidemiological study investigating the bidirectional risk association between AIBD and MG, although potentially impacted by possible limitations (eg, mislassification errors, restricted data points pertaining to disease characteristics/severity, and the possibility to perform muliple hypothesis tests leading to type 1 errors), suggests that clinicians should be aware of the increased risk for the development of MG during the clinical course of patients with AIBD. Given the potential implications of MGUS progression, closer clinical surveillance in AIBD patients may be warranted to ensure timely detection and management of plasma cell dyscrasias. Further studies are warranted to elucidate the underlying biological mechanisms and to better understand the prognosis of AIBD patients with MG.

## Declaration of generative AI and AI-assisted technologies in the writing process

During the preparation of this work, the authors used GPT4o (OpenAI, San Francisco, CA, USA) to improve readability in some sections. After using this tool, the authors reviewed and edited the content as needed and take full responsibility for the content of the publication.

## Conflicts of interest

Dr Ludwig has received travel funding from TriNetX. Drs Curman, Ngo, and Kasperkiewicz have no conflicts of interest to declare.
